# Dealing with missing data in a multi-question depression scale: a comparison of imputation methods

**DOI:** 10.1186/1471-2288-6-57

**Published:** 2006-12-13

**Authors:** Fiona M Shrive, Heather Stuart, Hude Quan, William A Ghali

**Affiliations:** 1 Department of Community Health Sciences, Faculty of Medicine, University of Calgary, Alberta, Canada; 2 Department of Medicine, Faculty of Medicine, University of Calgary, Alberta, Canada; 3 Centre for Health and Policy Studies, Faculty of Medicine, University of Calgary, Alberta, Canada; 4 The Centre for the Advancement of Health, Calgary Health Region, Calgary, Alberta, Canada; 5 Department of Community Health Sciences, Faculty of Medicine, Queen's University, Kingston, Ontario, Canada

## Abstract

**Background:**

Missing data present a challenge to many research projects. The problem is often pronounced in studies utilizing self-report scales, and literature addressing different strategies for dealing with missing data in such circumstances is scarce. The objective of this study was to compare six different imputation techniques for dealing with missing data in the Zung Self-reported Depression scale (SDS).

**Methods:**

1580 participants from a surgical outcomes study completed the SDS. The SDS is a 20 question scale that respondents complete by circling a value of 1 to 4 for each question. The sum of the responses is calculated and respondents are classified as exhibiting depressive symptoms when their total score is over 40. Missing values were simulated by randomly selecting questions whose values were then deleted (a missing completely at random simulation). Additionally, a missing at random and missing not at random simulation were completed. Six imputation methods were then considered; 1) multiple imputation, 2) single regression, 3) individual mean, 4) overall mean, 5) participant's preceding response, and 6) random selection of a value from 1 to 4. For each method, the imputed mean SDS score and standard deviation were compared to the population statistics. The Spearman correlation coefficient, percent misclassified and the Kappa statistic were also calculated.

**Results:**

When 10% of values are missing, all the imputation methods except random selection produce Kappa statistics greater than 0.80 indicating 'near perfect' agreement. MI produces the most valid imputed values with a high Kappa statistic (0.89), although both single regression and individual mean imputation also produced favorable results. As the percent of missing information increased to 30%, or when unbalanced missing data were introduced, MI maintained a high Kappa statistic. The individual mean and single regression method produced Kappas in the 'substantial agreement' range (0.76 and 0.74 respectively).

**Conclusion:**

Multiple imputation is the most accurate method for dealing with missing data in most of the missind data scenarios we assessed for the SDS. Imputing the individual's mean is also an appropriate and simple method for dealing with missing data that may be more interpretable to the majority of medical readers. Researchers should consider conducting methodological assessments such as this one when confronted with missing data. The optimal method should balance validity, ease of interpretability for readers, and analysis expertise of the research team.

## Background

Missing data are a common challenge in health research, and the problem is often pronounced in studies that use self-report instruments. As part of an outcome study in surgical patients, we measured levels of depression in surgical patients using a validated instrument, the Zung Self-rated Depression Scale (SDS) (Table 1)[1]. Among the 1931 patients surveyed, 351 did not fully complete the instrument. The quantity of missing data among those 351 subjects occasionally involved only 1 missing response in the entire instrument, with the majority of respondents missing 4 or less items. The remaining 1580 participants of the study completed all 20 questions of the SDS. Such missing data scenarios leave researchers with the choice of dropping cases entirely when they have missing responses to some questions, or alternatively, finding an imputation solution to deal with missing information.

**Table 1:** The Zung Self-rating Depression Scale (SDS)

	None or a little of the time	Some of the time	Good part of the time	Most of the time
1. I feel down-hearted, blue, and sad.	1	2	3	4
2. Morning is when I feel the best.	4	3	2	1
3. I have crying spells or feel like it.	1	2	3	4
4. I have trouble sleeping through the night.	1	2	3	4
5. I eat as much as I used to.	4	3	2	1
6. I enjoy looking at, talking to, and being with attractive men/women.	4	3	2	1
7. I notice that I am losing weight.	1	2	3	4
8. I have trouble with constipation.	1	2	3	4
9. My heart beats faster than usual.	1	2	3	4
10. I get tired for no reason.	1	2	3	4
11. My mind is as clear as it used to be.	4	3	2	1
12. I find it easy to do the things I used to.	4	3	2	1
13. I am restless and can't keep still.	1	2	3	4
14. I feel hopeful about the future.	4	3	2	1
15. I am more irritable than usual.	1	2	3	4
16. I find it easy to make decisions.	4	3	2	1
17. I feel that I am useful and needed.	4	3	2	1
18. My life is pretty full.	4	3	2	1
19. I feel that others would be better off if I were dead.	1	2	3	4
20. I still enjoy the things I used to do.	4	3	2	1

Scoring – The total score is calculated by adding the responses to the 20 questions. The maximum total is 80 (20 statements with a highest possible score of 4 each). The score is then converted into a total out of 100 by dividing the respondent's sum by 0.8. The cut points for the scale are:

(1) < 50 : normal range

(2) 50 – 59 : minimal or mild depression

(3) 60 – 69 : moderate to marked depression

(4) > 70 : severe depression

To gain insights into how to deal with missing data in such scenarios, we conducted a methodological study for which we selected the subset of participants with complete responses in the above-mentioned study, and simulated missing data scenarios by deleting observations. We then compared 6 methodological approaches to imputing replacement values for the deleted observations and assessed the accuracy of each of the six methods. These 6 methodological approaches are described in detail in the Methods section. Five missing data simulations were produced from a complete data set to permit this methodological comparison. Initially, data were deleted randomly with missing data probabilities of 10%, 20% and 30% applied equally to all questions on the Zung SDS. We then implemented a deletion algorithm pattern that assigned a higher probability of missing data to one particular question, a pattern of missing data that resembles that of our surgical outcome study. We also considered a scenario where the probability of missing was linked to patient characteristics (age and gender). Lastly, a missing not at random simulation was completed by linking the probability of missing one particular question to the response of that question. Our methodological approach and findings will inform researchers who encounter such missing data scenarios in the conduct of health research.

## Methods

A total of 1931 patients seen in the pre-operative assessment clinic of a tertiary care centre in Calgary, Alberta, Canada agreed to participate in the survey portion of a surgical outcomes study. After informed consent was obtained, participants were given a study package containing an introductory letter and the questionnaires. All questionnaires were self-reported and returned to the research assistant upon completion. Ethical approval was obtained from the University of Calgary Ethics Review Board.

The SDS questionnaire is a 20 question scale for which details are shown in Table 1. Each question is scored between 1 and 4, and a sum of responses is calculated. A previous version of the Zung SDS included 25 questions with a maximum total score of 100 [2]. To maintain comparability across the previous and the current version of the SDS instrument, the score from the current version is converted onto a 100-point scale. Thus, the calculated sum of scores across the 20 questions is converted to a 100-point scale by dividing the sum by 0.8. Respondents are classified as exhibiting depressive symptoms when their converted score is over 50.

As mentioned earlier, 1580 patients completed all items of the SDS questionnaire. Missing values were simulated in these complete cases by assigning each observation a number between 0 and 1 randomly selected from the uniform distribution (0,1); each number between 0 and 1 has an equal probability of being assigned. The assigned value was then used to assign missing values to selected observations. Initially, three missing completely at random (MCAR) scenarios were simulated; the probability of missing is not linked to any other patient characteristics. Observations assigned a value of less than 0.10 were deleted simulating a study with 10% of the collected data missing. For subsequent MCAR simulations the value was increased to 0.20, and then to 0.30. Subjects with no deleted values were then removed from the analysis since there is no missing value to impute.

We also considered an unbalanced MCAR scenario where the probability of missing question 6 ("I enjoy looking at, talking to, and being with attractive men/women" [Table 1]) was 20% and 10% for all other questions. This was done to mimic the pattern the missing data seen in our cohort where question 6 was found to be missing approximately twice as often as other questions among the incomplete cases. This simulation is referred to as the "Q6" simulation.

Next, a missing at random (MAR) simulation was completed; the probability of missing is linked to known patient characteristics. The probability of an observation being missing was linked to the age and gender of the patient. This association has been demonstrated in the literature with females and those over 65 being more likely to have missing values [3]. Thus, females over 65 were assigned a 20% probability of non-response; all other patients were assigned a 10% probability.

Lastly, a missing not at random simulation was completed (MNAR). In this scenario, the probability of missing depends on an unknown patient characteristic. All questions, except question 6, were assigned a probability of missing of 10%. If the response to question 6 was a 1 or 2, the probability of missing for question 6 was 5%. If the response was a 3 or a 4 the probability of missing was 20%. Thus, the probability of missing questions 6 was linked to the response of question 6 itself (an unknown characteristic in real missing data situations).

Six methods of imputation were compared: 1) random selection, 2) preceding question, 3) question mean, 4) individual mean 5) single regression and 6) a multiple imputation (MI) algorithm. Each method is briefly described below:

1) Random Selection

The imputed value was a randomly selected value from 1 to 4. This method was included to provide an example of an imputation method for which no participant characteristics are considered.

2) Preceding Response

The SDS questionnaire contains a series of questions that when asked together assess the subject's depressive symptoms. Subjects tend to respond at levels consistent with their mental state throughout the instrument. In such situations, a subject's response to the preceding question could be used as a source of information for determining the missing response. For this method, we replicated the preceding question's response to impute the missing response.

3) Question Mean

The question mean method imputes the overall mean of the specific question from the entire cohort. For example, if a participant has a missing value for question 17, the imputed value is the mean calculated from the completed question 17 for the entire cohort.

4) Individual Mean

Individual mean imputation can also be used as a simple form of imputation in such scenarios. The imputed value is the calculated mean of a given subject's complete responses to other questions. If a participant has 2 missing responses, the values are filled with the calculated average of the remaining completed 18 questions.

5) Single Regression

A regression model was built to predict the missing value based on cases with complete data. The missing value was considered the outcome variable with all other available data points for an individual used as the predictor variables. Since traditional regression approach would result in a different model for each pattern of missing data, we applied the multiple regression procedure from SAS (see below) using only one repetition.

6) Multiple Imputation (MI)

The experimental version of MI available in SAS 8.1 was used [4]. The method is based on Rubin's work [5] that attempts to estimate a missing value with a plausible set of values. The method assumes a multivariate normal distribution and that the missing data are MAR. The resulting statistics appropriately reflect the uncertainty in the data due to missing values. The imputation is carried out in three steps. The missing data are filled 5 times generating 5 complete unique data sets. Each data set is analysed separately to calculate a mean, and standard deviation. Then, the results from each analysis are combined to produce an overall mean and standard deviation for each missing value. The missing values are predicted based on a specified list of characteristics that are used as predictors of the missing value(s). In our case, the predicting variables used in the MI procedure to predict missing responses were the responses to completed questions.

## Analysis

For each method, the SDS score was calculated first with imputed values, and then with the "true" values, that are known to be true because the missing values are artificially created. The sample mean and standard deviation SDS scores were compared to the known population statistics (the latter derived from the known values prior to creation of missing values). The Spearman correlation coefficient, the percent of patients misclassified as depressive, and the Kappa statistic for dichotomous classification of depression were also calculated for each method. All three of these calculated statistics, through differing approaches, represent the level of agreement between the imputed values and the known "true" values. The Spearman correlation coefficient is a non-parametric statistic based on the ranks of the observations. The Kappa statistic expresses the amount of agreement (over and above that expected due to chance alone) between the dichotomous assessments of depression (present vs. absent) that are based on the imputed and observed SDS scale scores. Landis and Koch categorize Kappa into five categories: less than 0.2 indicating "poor agreement", 0.21 to 0.40 indicating "fair agreement", 0.41 to 0.60 indicating "moderate agreement, 0.61 to 0.80 indicating "substantial agreement" and greater than 0.81 indicating "near perfect agreement"[6].

## Results

Table 2 displays the distribution of randomly deleted responses for each of the missing patterns that we created (p = 0.10, p = 0.20, p = 0.30, "Q6", "MAR – age and sex" and "MNAR"). As the probability of a missing value increases, the average number of missing observations per participant increases. At a probability of 10% (p = 0.10) the majority of participants have three or less observations artificially missing. When the probability of missing is increased to 30% (p = 0.30), the majority of participants have between four and seven observations randomly deleted.

**Table 2:** Distribution of randomly deleted missing responses (N = 1580)

**Missing Data Scenario**	**P = 0.10**		**P = 0.20**		**P = 0.30**		**Q6**		**MAR – Age and Sex**		**MNAR**	
Total missing responses	N	% of total	N	% of total	N	% of total	N	% of total	N	% of total	N	% of total
0	201	12.7	18	1.1	174	11.0	1	0.1	151	9.6	174	11.0
1	422	26.7	83	5.3	373	23.6	7	0.4	376	23.8	422	26.7
2	432	27.3	220	14.0	463	29.3	42	2.7	435	27.5	466	29.5
3	306	19.4	341	21.6	305	19.3	141	8.9	317	20.1	315	19.9
4	147	9.3	314	19.9	161	10.2	226	14.3	172	10.9	122	7.7
5	52	3.3	296	18.7	85	5.4	288	18.2	78	4.9	59	3.7
6	18	1.1	171	10.8	12	0.8	305	19.3	35	2.2	15	1.0
7	2	0.1	92	5.82	6	0.4	241	15.3	11	0.7	5	0.3
8	0	0	34	2.2	1	0.1	158	10.0	3	0.2	2	0.1
9	0	0	9	0.6	0	0	90	5.7	2	0.1	0	0
10	0	0	0	0.0	0	0	46	2.9	0	0	0	0
11	0	0	1	0.1	0	0	25	1.6	0	0	0	0
12	0	0	1	0.1	0	0	6	0.4	0	0	0	0
13	0	0	0	0	0	0	2	0.1	0	0	0	0
14	0	0	0	0	0	0	2	0.1	0	0	0	0

Table 3 presents the mean, standard deviation, Spearman correlation coefficient, percent misclassified and kappa statistic for each imputation method. When the data have a low percentage of missing values (p = 0.10), individual mean, question mean, preceding question, single regression and MI all produce a Kappa statistic greater than 0.81 indicating "near perfect agreement." Random selection does not perform as well with a Kappa statistic of 0.684. The calculated mean for both random selection (45.99) and preceding question (44.69) is significantly different than the population mean (43.68) while the question mean produces a significantly different standard deviation (9.84) from the population value (10.98). MI produces the most valid imputed values with the highest Kappa statistic (0.893), lowest percent misclassified (4.7%) and neither the calculated mean value nor standard deviation significantly differing from the known population values. However, notably, the individual mean does produce similar statistics.

**Table 3:** Diagnostic measures for imputation methods

Missing Data Scenario	Method	Mean	SD	Spearman	% Misclassified	Kappa
**P = 0.10 ** N = 1379** μ = 43.68 σ = 10.98	Random Selection	45.99*	10.65	0.906	15% (207)	0.684
	Preceding Question	44.69*	10.07	0.946	8.7% (120)	0.807
	Question Mean	43.75	9.84*	0.986	7.5% (104)	0.823
	Individual Mean	43.74	11.11	0.986	5.4% (74)	0.880
	Single Regression	44.03	10.71	0.981	5.6%(77)	0.873
	Multiple Imputation	44.01	10.73	0.987	4.7% (65)	0.893

**P = 0.20** N = 1562** μ = 43.64 σ = 10.98	Random Selection	47.25*	11.14	0.784	28.2% (440)	0.452
	Preceding Question	46.41*	9.79*	0.898	14.4% (225)	0.700
	Question Mean	43.59	8.88*	0.974	12.1% (189)	0.709
	Individual Mean	43.59	11.26	0.974	8.9% (139)	0.802
	Single Regression	44.03	10.65	0.966	9.6% (150)	0.781
	Multiple Imputation	44.06	10.49	0.976	7.0% (110)	0.839

**P = 0.30** N = 1579** μ = 43.62 σ = 10.93	Random Selection	49.09*	11.92*	0.610	41.0% (647)	0.267
	Preceding Question	48.62*	9.55*	0.867	23.6% (373)	0.549
	Question Mean	43.60	8.05*	0.958	14.9% (235)	0.629
	Individual Mean	43.66	11.33	0.955	10.8% (171)	0.760
	Single Regression	44.39	10.33	0.937	11.4%(180)	0.738
	Multiple Imputation	44.32	10.21	0.959	9.2% (145)	0.789

**Q6** N = 1406** μ = 43.49 σ = 10.89	Random Selection	45.62	10.38	0.901	16.6% (233)	0.649
	Preceding Question	41.66*	10.73	0.970	10.2% (143)	0.753
	Question Mean	43.43	9.67*	0.987	8.4% (118)	0.798
	Individual Mean	43.37	11.03	0.984	5.7% (80)	0.870
	Single Regression	43.66	10.67	0.978	6.8%(95)	0.842
	Multiple Imputation	43.67	10.61	0.986	5.8% (81)	0.866

**MAR – Age and Sex** N = 1429** μ = 43.60 σ = 10.90	Random Selection	45.85	10.48	0.885	18.1 %(259)	0.618
	Preceding Question	44.81	10.09	0.940	8.9% (127)	0.804
	Question Mean	43.63	9.65*	0.984	7.4% (106)	0.825
	Individual Mean	43.65	11.05	0.982	5.7% (82)	0.867
	Single Regression	43.89	10.67	0.978	7.1%(102)	0.835
	Multiple Imputation	43.91	10.58	0.985	5.3% (77)	0.877

**MNAR** N = 1406** μ = 43.51 σ = 10.80	Random Selection	45.82*	10.46	0.899	15.7% (221)	0.741
	Preceding Question	44.44	10.06	0.947	9.7% (136)	0.839
	Question Mean	43.51	9.66*	0.987	8.4% (118)	0.850
	Individual Mean	43.50	10.90	0.985	5.9% (83)	0.902
	Single Regression	43.54	10.78	0.975	7.7% (108)	0.871
	Multiple Imputation	43.54	10.65	0.986	6.1% (86)	0.897

* significant difference from the population statistics at 95% confidence

** Participants for which no observations were randomly deleted are excluded from the analysis. When there are no missing values to impute, the calculated score is the same as the known "true" score thus the scores correlate perfectly (spearman = 1.0)

**Figure 1:**
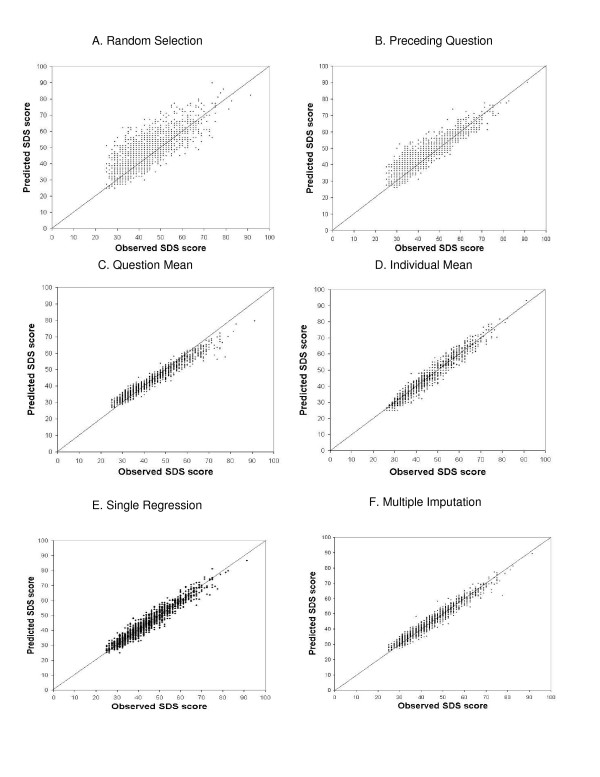
Predicted versus observed scores for each imputation technique with a probability of missing of 20%.

Figure 1 graphically displays the correlation between the predicted values and the observed scores when the probability of missing data is increased to 20%. Both MI and single regression (panels E and F) show a tight cluster of observations around the line of agreement (the diagonal line). Individual mean imputation (panel D) portrays a slightly more scattered distribution about the agreement line but maintains a fairly tight distribution. The resulting scatter from the other three methods is more dispersed with random selection (panel A) producing a number of observations that fall far away from the diagonal line. A slight rotation away from the diagonal line of agreement (and towards a horizontal line) is observed in the question mean imputation (panel C). All the other methods produce scatter patterns that fall about the diagonal line of agreement (a straight line with a slope of 1).

As the percent of missing information is increased further (p = 0.20 and p = 0.30 simulations), multiple imputation becomes increasingly more accurate than the other five methods (Table 3). The kappa statistic remains high when the probability of a missing value is increased to 30% (p = 0.30) indicating that MI is an appropriate imputation method even when dealing with substantial missing data. Individual mean imputation also continues to perform reasonably well. The imputed mean and standard deviation do not differ significantly from the population statistics. The Kappa statistic remains high dropping only to 0.760 – still within the range of "substantial agreement." However, the percent misclassified for depression status increases to approximately 10%. Similar statistics are seen with single regression imputation (Kappa 0.738, percent misclassified 11.4%).

The question mean method and preceding question method yield similar percentages of misclassified observations and steady declines in the Kappa statistic as the probability of missing data increases from 0.10 to 0.30. The standard deviation resulting from both methods differs significantly from the population value. The calculated values are significantly lower than the observed values indicating an underestimation of the standard deviation.

As expected, the random selection method produces poor correlations with an increase in the probability of missing data. At a probability of 30%, the Kappa statistic has dropped to 0.267 indicating "fair agreement" and the percent of misclassified observations has increased to 41%, nearly half of the observations.

The unbalanced MCAR and MAR (Q6, and MAR-age and sex) and MNAR simulations produced similar results to the 10% probability of missing data simulation. The underlying assumption of the MI method – that the missing data are MAR – is not satisfied in the latter of these scenarios. Thus, MI tends to perform less well in such situations, and in our analysis, the individual mean method actually outperforms MI in the unbalanced MCAR (Q6) simulation and the MNAR simulation. The other five methods produce approximately the same statistics as the 10% MCAR missing data pattern.

Table 4 demonstrates how agreement measures change as individual subjects have increasing missing items in the 20% MCAR (p = 0.20) missing data scenario. The Spearman correlation values and Kappa values drop as the number of missing items increases. This drop is quite marked for the random selection method. In contrast, the drop is minimal for the multiple imputation method, single regression and individual mean methods indicating that all three methods are more robust than the question mean, preceding question and random selection method.

**Table 4:** Correlation with increased missing items in the P = 0.20 missing data scenario.

**Method of Imputation**	**Random Selection**		**Preceding Question**		**Question Mean**		**Individual Mean**		**Single Regression**		**Multiple Imputation**	
# of Missing Items (P = 0.20)	Spearman	Kappa	Spearman	Kappa	Spearman	Kappa	Spearman	Kappa	Spearman	Kappa	Spearman	Kappa
1 (N = 83)	0.983	0.971	0.983	0.910	0.993	1.000	0.992	0.971	0.990	0.970	0.993	1.000
2 (N = 220)	0.941	0.605	0.961	0.741	0.987	0.804	0.987	0.794	0.984	0.783	0.988	0.841
3 (N = 341)	0.877	0.563	0.944	0.791	0.981	0.771	0.980	0.833	0.974	0.832	0.982	0.848
4 (N = 314)	0.816	0.454	0.916	0.739	0.976	0.743	0.973	0.823	0.967	0.764	0.974	0.854
5 (N = 296)	0.783	0.438	0.930	0.661	0.978	0.641	0.977	0.780	0.969	0.784	0.978	0.853
6 (N = 171)	0.618	0.182	0.890	0.569	0.963	0.651	0.953	0.745	0.934	0.710	0.957	0.805
7 (N = 92)	0.508	0.114	0.791	0.454	0.948	0.346	0.930	0.630	0.918	0.600	0.948	0.649
8 (N = 34)	0.563	0.191	0.848	0.698	0.970	0.401	0.958	0.827	0.943	0.884	0.978	0.831

Recognising that there is inherent uncertainty relating to imputed values, we also assessed the number of times that the "true" value was captured by the range of estimated values produced by the MI procedure with 5 imputations (Table 5). We assessed this for three different missing data scenarios (MCAR-p = 0.10, MAR-age and sex and MNAR). For comparison, we also assessed the percentage of the time that the imputed value produced by single regression imputation agreed with the true value. Table 5 reveals that the range of imputed values in MI encompassed the true values anywhere from 77% to 98% of the time across the 20 questions, whereas the imputed values in single regression matched the true values far less often.

**Table 5:** Comparison of MI and single regression in the "capture" of the true values using 3 missing data scenarios

	**MCAR – P = 0.10 N = 1379**			**MAR – Age and Sex N = 1429**			**MNAR N = 1406**		
Question	# cases missing N	MI coverage of true value by range N (%)	Single regression agreement with true value N (%)	# cases missing N	MI coverage of true value by range N (%)	Single regression agreement with true value N (%)	# cases missing N	MI coverage of true value by range N (%)	Single regression agreement with true value N (%)
1	150	142 (95%)	88 (59%)	189	173 (92%)	115 (61%)	139	124 (89%)	76 (55%)
2	175	141 (81%)	48 (27%)	182	142 (78%)	46 (25%)	161	124 (77%)	46 (29%)
3	155	150 (97%)	111 (72%)	173	164 (95%)	114 (66%)	156	146 (94%)	94 (60%)
4	146	115 (79%)	47 (32%)	176	137 (78%)	47 (27%)	170	143 (84%)	40 (24%)
5	158	125 (79%)	47 (30%)	185	150 (81%)	45 (24%)	159	126 (79%)	49 (31%)
6	160	132 (83%)	43 (27%)	170	132 (78%)	44 (26%)	249	206 (83%)	75 (30%)
7	145	132 (91%)	64 (44%)	199	181 (91%)	110 (55%)	167	153 (92%)	85 (51%)
8	161	142 (88%)	58 (36%)	190	171 (90%)	82 (43%)	152	132 (87%)	70 (46%)
9	172	164 (95%)	94 (55%)	184	172 (93%)	107 (58%)	177	164 (93%)	93 (53%)
10	174	156 (90%)	69 (40%)	166	144 (87%)	65 (39%)	152	131 (86%)	64 (42%)
11	163	126 (77%)	63 (39%)	184	153 (83%)	50 (27%)	142	119 (84%)	49 (35%)
12	151	126 (83%)	66 (44%)	168	146 (87%)	60 (36%)	155	131 (85%)	52 (34%)
13	151	135 (89%)	55 (36%)	155	135 (87%)	71 (46%)	141	125 (89%)	53 (38%)
14	165	149 (90%)	74 (45%)	195	175 (90%)	84 (43%)	153	134 (88%)	68 (44%)
15	157	134 (85%)	53 (34%)	186	169 (91%)	63 (34%)	147	126 (86%)	60 (41%)
16	147	132 (90%)	59 (40%)	194	154 (79%)	72 (37%)	163	141 (87%)	59 (36%)
17	152	143 (94%)	87 (57%)	184	179 (97%)	105 (57%)	150	135 (90%)	81 (54%)
18	153	135 (88%)	68 (44%)	169	154 (91%)	93 (55%)	152	140 (92%)	84 (55%)
19	177	174 (98%)	142 (80%)	174	167 (96%)	135 (78%)	169	164 (97%)	147 (87%)
20	162	140 (86%)	73 (45%)	185	157 (85%)	75 (41%)	169	152 (90%)	72 (43%)

## Discussion

Multiple imputation is the most accurate imputation method in four of the six scenarios that we assessed in our SDS questionnaire dataset. However, individual mean imputation, a simple imputation method, performs almost as well as MI, providing means and standard deviations for the population depression scores that closely approximate the known population values in all simulations. Interestingly, in three of our unbalanced simulations, individual mean imputation actually performs slightly better than multiple imputation. This implies that there may be situations where multiple imputation is not the optimal imputation method.

Although multiple imputation is probably the most accurate and valid imputation method, it has several disadvantages. The method itself is complex and utilises advanced statistical modeling that is likely to be unfamiliar to many readers and researchers; advanced statistical expertise is required to implement the method with confidence.

Our single regression imputation method applied the same technique as the multiple imputation method with one iteration instead of the five used for multiple imputation. Thus, the differences seen between those two methods are due to the repetition of the process. The difference in the percent misclassified is notable suggesting that repetition is an important component if regression is to be used to impute values.

In contrast, individual mean imputation is simpler and thus likely to be understood by a larger proportion of medical readers. Indeed, it is a more intuitive approach to imputing values. The underlying assumption is simply that a respondent will have similar responses throughout the questionnaire – a reasonable assumption for an ordinally-scaled instrument like the SDS[7]. In our case, individual mean imputation produces excellent correlation coefficients and valid imputation values.

An intriguing tilt away from the diagonal line of agreement is noted in the scatter plot for the question mean method presented in Figure 1, Panel C. This presumably arises because patients with low observed depression scores receive a higher imputed score drawn from the mean question score seen in the entire population. The result is a slight overestimation of those individuals' depression scores. The same phenomenon occurs at the opposite end of the distribution of depression scores. Patients with high observed scores (prior to the missing data simulation) are assigned a lower imputed score that reflects the mean score for that question in the entire population. This results in an underestimation of those individuals' depression scores. This combination of overestimation of scores in patients with low depression scores, and underestimation of scores in those with high scores, leads to the observed 'rotation' of the scatter pattern seen in Figure 1, panel C. Such rotation is not observed in any of the other methods.

While the conclusions regarding 'best' solutions to our particular missing data problem are relatively clear, our findings may not be applicable to other missing data scenarios. Other databases and survey questionnaires may have characteristics that would yield different findings and conclusions regarding the optimal missing data solution. We therefore encourage readers to simply view our evaluative work as a template for methodological evaluation of potential missing data solutions in other datasets. We encourage other researchers to follow a similar approach to the one presented here for conducting an assessment of possible imputation methods.

Our findings resemble those of Gmel [8] and Hawthorne and Elliot [9] who assessed the performance of various imputation methods for dealing with missing responses in questionnaires. Both of these studies demonstrated that although sophisticated imputation methods such as "hot-deck imputation" have advantages, that simple single-value imputation methods also perform very well. Others have cautioned that, although mathematically simpler, mean imputation can lead to underestimation of the variance within the data and techniques such as multiple imputation should be used [10]. Farclough and Cella [11] demonstrated strong performance of a simple imputation method that resembles our 'individual mean' imputation approach, and found that this approach is superior to simple case deletion or a number of other simple imputation methods. Fayers and colleagues [12], however, remind us that although simple imputation methods often perform quite well, that there are some cautions to their widespread use. They go on to provide a useful checklist that researchers should apply when considering the use of simple imputation methods for missing data scenarios.

Our study demonstrates the use of multiple imputation to derive a single value – the missing observation for a specific respondent. Multiple imputation can also be applied in different ways, perhaps most typically to estimate the parameters of a regression model in the context of multivariable analyses. The latter approach typically provides estimates of β coefficients that accurately reflect the uncertainty due to missing values. Table 5 also reveals that multiple imputation more fully characterizes the uncertainty inherent in imputed data. Our use of multiple imputation to determine population mean values is thus not the only approach to using the method. Nevertheless, the generally favourable performance of the SAS multiple imputation procedure in this analysis speaks to its general versatility and applicability in this context.

It has been suggested that MI should only be used in circumstances where the data are MAR[13]. A corollary is that MI would not perform well in circumstances where the pattern of missing data is not MAR or MCAR (a common situation in applied research). Our results actually demonstrate a reasonably strong performance of MI in not only MAR and MCAR scenarios, but also in the MNAR simulation. In support of our finding, Schafer has argued that, when applied in rich datasets, bias resulting from the violation of the MAR assumption required for multiple imputation can be minimized [14]. Similarly, Faris et al. have empirically demonstrated strong performance of multiple imputation in a dataset with a MNAR pattern of missing data [15]. Our results agree with the latter finding and perhaps indicate that the MAR assumption may not be an absolute prerequisite for MI. In this regard, further investigation of MI methodologies in real or simulated MNAR situations is warranted.

Our study also has caveats and limitations. The random simulations carried out may not be reflective of the patterns of missing data seen in real situations. Furthermore, while the Zung SDS may be particularly suited to the six types of imputation explored here, other imputation methods may be more appropriate for other questionnaires. Since the Zung has 20 questions that are all measuring the same construct, the instrument should be robust to the presence of missing values. Thus, individual mean imputation is particularly suited to this scale. Other scales and instruments may not be as amenable to the imputation methods presented here.

Lastly, for the purposes of this methodological study on imputation methods, we excluded the 351 participants of our surgical outcomes study who had truly incomplete SDS questionnaires. In the eventual application of imputation methods to these real missing data cases in our surgical outcome study, we will never really know how well our imputed values represent the values that might have been provided had a response item not been missing. In this regard, our methodological comparison of imputed values against artificially created missing values does not reflect applied imputation scenarios, where a researcher never really knows how well the imputed values represent what data values would have been had they been present.

## Conclusion

In our data analysis scenario, multiple imputation was usually the most accurate method of imputation. Notably, however, a simpler method – individual mean imputation – demonstrated comparable accuracy in three MCAR scenarios and actually performed slightly better than multiple imputation in three scenarios where data were missing in an unbalanced manner. For our missing data problem, we therefore conclude that individual mean imputation is the best approach for our work, because it provides an attractive balance of both accuracy and conceptual simplicity. Ultimately, however, the optimal imputation method in any situation should be selected based on a balance of the statistical expertise of the research team, validity of the method, and ease of interpretability for readers. We encourage researchers to conduct similar methodological assessments to find the most suitable method of imputation for their specific datasets and measures.

## Competing interests

The author(s) declare that they have no competing interests.

## Authors' contributions

FS carried out the statistical analysis and drafted the manuscript. HS lead the Surgical Outcomes Study participating the conception, design and coordination of the study. HQ managed the Surgical Outcomes database. WG oversaw the Surgical Outcomes Study and conceived, designed and oversaw the current study. All authors read and approved the final manuscript.

## Pre-publication history

The pre-publication history for this paper can be accessed here:


